# Supplementary material for: Association between median household income, state Medicaid expansion status, and COVID-19 outcomes across US counties
Source: PLoS One. 2022 Aug 11;17(8):e0272497. doi: 10.1371/journal.pone.0272497 (PMC9371257; doi:10.1371/journal.pone.0272497)
Supplement: S1 Table — (DOCX) [file pone.0272497.s001.docx]

| Supplemental Table 1. List of Medicaid Expansion and Non-Medicaid Expansion states as of January 1, 2020 |
| --- |

| **Medicaid Expansion States**  **n = 37** | **Medicaid Non-Expansion States**  **n = 14** |
| --- | --- |
| Alaska | Alabama |
| Arizona | Florida |
| Arkansas | Georgia |
| California | Kansas |
| Colorado | Mississippi |
| Connecticut | Missouri |
| Delaware | North Carolina |
| Federal District of Columbia | Oklahoma |
| Hawaii | South Carolina |
| Idaho | South Dakota |
| Illinois | Tennessee |
| Indiana | Texas |
| Iowa | Wisconsin |
| Kentucky | Wyoming |
| Louisiana |  |
| Maine |  |
| Maryland |  |
| Massachusetts |  |
| Michigan |  |
| Minnesota |  |
| Montana |  |
| Nebraska |  |
| Nevada |  |
| New Hampshire |  |
| New Jersey |  |
| New Mexico |  |
| New York |  |
| North Dakota |  |
| Ohio |  |
| Oregon |  |
| Pennsylvania |  |
| Rhode Island |  |
| Utah |  |
| Vermont |  |
| Virginia |  |
| Washington |  |
| West Virginia |  |
