# Supplementary material for: Association between median household income, state Medicaid expansion status, and COVID-19 outcomes across US counties
Source: PLoS One. 2022 Aug 11;17(8):e0272497. doi: 10.1371/journal.pone.0272497 (PMC9371257; doi:10.1371/journal.pone.0272497)
Supplement: Attachment — Submitted filename: plos one review comments-plos one.docx [file pone.0272497.s002.docx]

Author’s Reply to Reviewer’s Comments

Major Comments:

1/ The paper concludes that health care coverage in Medicaid-expansion states is most likely to be the main factor that explains the differences in mortality between the income quartiles. However, the estimates are too large for this to be credible. For example, there is a 41% increase in mortality between the 1st quartile and the 4th quartile in Medicaid non-expansion states. Given that roughly 80% of COVID-19 related deaths come from individuals aged 65 and above (https://www.cdc.gov/nchs/nvss/vsrr/covid_weekly/index.htm#SexAndAge), who are on Medicare and therefore not affected by the Medicaid expansion, this is implausible.

R/ We agree with the reviewer and have toned down our discussion to state that the mechanisms are likely multifactorial (second and third paragraphs, discussion). We also changed	our conclusion to “*Future studies are needed to untangle which state policies have the most impact in the attenuation of the excessive COVID-19 mortality risk associated with socioeconomically disadvantaged communities.”*

2/ Relating to the previous comment, a far more likely explanation is that there are other unobserved factors that influence income, Medicaid expansion status, and COVID-19 mortality, leading to omitted variable bias and misleading conclusions. For instance, state politics are widely known to be the dominant factor influencing state Medicaid expansion status, and this also affects individuals’ perception of the pandemic and their response when they get sick. A person who do not believe that COVID is a major risk is more likely to become infected with COVID-19 and also less likely to seek medical help when presented with COVID symptoms. Differences in social distancing/mask wearing behavior by political views are now widely documented in the literature, and these almost certainly lead to a biased estimate for the effect of Medicaid expansion.

R/ We agree with the reviewer (please see response to comment 1). We added in the discussion (third paragraph) that: *“It is possible that Medicaid expansion is only a marker of the state-level policy towards COVID-19, in terms of mask and social distancing mandates as well as health education and promotion practices for the population, all of which could influence population behavior…”.* However, we also discussed that since there was no significant difference in COVID-19 incidence across income quartiles, the observed mortality outcome differences across income quartiles in non-Medicaid expansion states can also be related to a lower health care access due to lack of insurance after contracting COVID-19, which may lead to delay in seeking care, less follow-up post care, leading to worse COVID-19 outcomes. We also included the possibility of bias as a limitation in the discussion.

3/ One thing to note is that because the Medicaid expansion mostly affects the eligible population, which are young adults in the 1st income quartile (reported as $52,794 in Table 1), it is more intuitive to select the 1st quartile as the reference group instead of the 4th quartile. For the same reason, the discrepancies in mortality between the other quartiles and the 4th quartile are not likely to come from the increase in health coverage from the Medicaid expansion.

R/ We apologize for the lack of clarity. We have chosen quartile 4 as the referent because the population without health insurance, hence most vulnerable to COVID-19 complications, are mostly in the lowest income quartile (quartile 1, median household income $38,514.62, 14% without insurance, as compared to quartile 4, median household income $71,139.70, 9% without insurance, Table 1), a percentage that is much larger in non-Medicaid expansion states (8.7% vs. 15.2%, Table 3). Indeed, based on results in Table 4, most differences in mortality between Medicaid and non-Medicaid expansion states come from the lowest income quartiles (quartiles 1-3).

4/ The recent literature has shown that non-pharmacological interventions such as stay-at-home orders play a significant role in both COVID-19 incidence and mortalities. Leaving these factors out of the analyses will also create bias in the results (as they are also driven largely by politics).

R/ We agree with the reviewer and have included health education and promotion to promote behavior change, as well as state policies respect to mask and social distancing mandates in the discussion and conclusion statements (please see response to comments 1 and 2). We also included as a limitation in the discussion the following: *“We are aware that policies regarding social distancing and mask mandate may influence the outcomes, it is difficult to incorporate these into the analyses given the ever-changing nature of these policies through-out the year and the disparate execution of these mandates at the regional level. Instead, we used the cumulative outcome approach to study the Medicaid-expansion policy that was unaltered during 2020. Although some states adopted Medicaid expansion into their state constitution during 2020 (Missouri and Oklahoma), none of them achieved implementation stage during 2020.”*

5/ It is stated in the manuscript that data for the period from January 20 to December 6, 2020, were used in the analyses. However, it is unclear how the time dimension enters the analyses because in Table 2, the unit at the county level. Did the authors just use cumulative data from Dec 6 (which would be reflective of the entire year)?

R/ We apologize for the lack of clarity. We added in methods that we used the cumulative data from January 20 to December 6, 2020.

Minor Comments:
- For clarity, numbers should include thousand separators.

R/ Numbers are included now in thousand separators.

- Results on COVID-19 cases should also be presented for reference purposes (perhaps in the supplemental document), even if they are insignificant.

R/ Results on COVID-19 cases are now presented in the text and within the tables 2, 3 and 4.

- Interpretations of results are not clear in the manuscript. It will be more informative to readers if the authors include a designated paragraph that explain the estimates in Figure 1.

R/ The last paragraph and a new Table 4 are now added to explain the estimates in Figure 1.

We appreciate and thank the reviewer for his/her comments, to which we provided a point-to-point answer in the reply to reviewer’s document, all of which, made our paper a much improved one.

Wen-Chih Wu, MD
