# Supplementary material for: Association between median household income, state Medicaid expansion status, and COVID-19 outcomes across US counties
Source: PLoS One. 2022 Aug 11;17(8):e0272497. doi: 10.1371/journal.pone.0272497 (PMC9371257; doi:10.1371/journal.pone.0272497)
Supplement: Attachment — Submitted filename: Response to reviewers.docx [file pone.0272497.s004.docx]

Author’s Reply to Reviewer’s Comments

Journal Requirements:

1/ Please ensure that your manuscript meets PLOS ONE's style requirements, including those for file naming. The PLOS ONE style templates can be found at
<https://journals.plos.org/plosone/s/file?id=wjVg/PLOSOne_formatting_sample_main_body.pdf> and
<https://journals.plos.org/plosone/s/file?id=ba62/PLOSOne_formatting_sample_title_authors_affiliations.pdf>

**R/ We apologize for the not adhering by PLOS ONE's style requirements. We have altered the format of the manuscript to reflect the updated PLOS ONE's style requirements, including those for file naming.**

2/ Thank you for stating the following financial disclosure: Yes, this project is supported by the Department of Veterans Affairs, Health Services Research Award IRP-20-003 (WW, project PI). Please state what role the funders took in the study. If the funders had no role, please state: "The funders had no role in study design, data collection and analysis, decision to publish, or preparation of the manuscript." If this statement is not correct you must amend it as needed. Please include this amended Role of Funder statement in your cover letter; we will change the online submission form on your behalf.

**R/ We apologize for the omission regarding the role of the funders in the study. We have adjusted the financial disclosure statement to include a statement clarifying the role of the funders in the study.**

3/ Thank you for stating the following in the Acknowledgments/ Funding Section of your manuscript: "This project is supported by the Department of Veterans Affairs, Health Services Research Award IRP-20-003 (WW, Project PI)". Please note that funding information should not appear in the Acknowledgments section or other areas of your manuscript. We will only publish funding information present in the Funding Statement section of the online submission form. Please remove any funding-related text from the manuscript and let us know how you would like to update your Funding Statement. Currently, your Funding Statement reads as follows: "Yes, this project is supported by the Department of Veterans Affairs, Health Services Research Award IRP-20-003 (WW, project PI)" Please include your amended statements within your cover letter; we will change the online submission form on your behalf.

**R/ Thank you for bringing this to our attention. We have removed all information relating to the funding source in the Acknowledgments section and other areas of the manuscript. Additionally, we have updated the cover letter to include information regarding funding. We would like the online submission form to state the following: “This project is supported by the Department of Veterans Affairs, Health Services Research Award IRP-20-003 (WW, Project PI). The funders had no role in study design, data collection and analysis, decision to publish, or preparation of the manuscript.”**

4/ We noted in your submission details that a portion of your manuscript may have been presented or published elsewhere. Please clarify whether this publication was peer-reviewed and formally published. If this work was previously peer-reviewed and published, in the cover letter please provide the reason that this work does not constitute dual publication and should be included in the current manuscript.

**R/ While awaiting information a decision from PLOS ONE, the authors of the manuscript added portions of the manuscript to the preprint server ‘medRxiv’, an option endorsed by PLoS ONE. The preprints on medRxiv are initial reports, and as such, it is not peer-reviewed or considered formally published.**

5/ Please amend your list of authors on the manuscript to ensure that each author is linked to an affiliation. Authors’ affiliations should reflect the institution where the work was done (if authors moved subsequently, you can also list the new affiliation stating “current affiliation:….” as necessary).

**R/ We apologize for the oversight about the affiliations of the authors of the manuscript. We have amended the list of authors to reflect the authors affiliations during the time when the work was done.**

6/ Please include captions for your Supporting Information files at the end of your manuscript, and update any in-text citations to match accordingly. Please see our Supporting Information guidelines for more information: <http://journals.plos.org/plosone/s/supporting-information>

**R/ We have amended the in-text citations to match the PLOS ONE's style requirements. Also, the Supporting Information files are at the end of the manuscript.**

8/ Thank you for including your ethics statement on the online submission form: "Providence Veterans Affairs Medical Center Institutional Review Board, formal waiver of approval due to non-Human subject research, project 1660196." To help ensure that the wording of your manuscript is suitable for publication, would you please also add this statement at the beginning of the Methods section of your manuscript file.

**R/ We have revised the beginning of the Methods section of the manuscript to include the ethics statement from the Providence Veterans Affairs Medical Center Institutional Review Board.**

9. Please review your reference list to ensure that it is complete and correct. If you have cited papers that have been retracted, please include the rationale for doing so in the manuscript text, or remove these references and replace them with relevant current references. Any changes to the reference list should be mentioned in the rebuttal letter that accompanies your revised manuscript. If you need to cite a retracted article, indicate the article’s retracted status in the References list and also include a citation and full reference for the retraction notice.

**R/ We have reviewed the reference list to ensure that it is complete and correct. During our review, we did not come across any cited papers that have since been retracted.**

Author’s Responses to Reviewer's Questions:

Reviewer #1: I am satisfied by the revisions. In particular, the limitations to causal inference are now clearly acknowledged.

**R/ Thank you for taking the time to provide insight into our work.**

Reviewer #2: “Dear PONE Journal Team of editorials, I am thankful for the chance given to me to review the manuscript titled “Association between Median Household Income, State Medicaid Expansion Status, and COVID-19 Outcomes Across US Counties”. The article has benefit for measuring the association between income, medical expansion across different countries in US even if the spelling for countries is ‘counties’. Here by the way what does across the US countries mean? Is US one or more country with fifty plus states? Anyways, the following are my comments;”

**R/ We appreciate your review of our work. After thorough review of the manuscript, we were unable to identify any occurrences of the use of ‘countries’. The analysis included 3142 United States counties (a political and administrative division within the 50 states) including the District of Columbia.**

- “Is incidence and mortality the only outcome? Why didn’t you focus on it mainly? Why not risk of being infected by COVID 19? Then the continuum of illness through the diseases process? Why not recovery? What are the complications? Try to address multiple whys?”

**R/ We appreciate your review of our work. In order to avoid over-extending the scope of paper and distract the reader, we have chosen to focus only on two main COVID outcomes, the COVID incidence and COVID mortality. As such, the incident rate ratio reported in our results would be considered an epidemiologically acceptable estimate of the relative risk of being infected with COVID and the mortality risk ratio would be an estimate of the worst complication of COVID, i.e. death. We did not address the recovery portion of COVID since it would be outside the scope of the current paper.**

- “Is the median income an established/factful US income before or during the era of the pandemic? Why median? Why not high income? Why not low income?”

**R/ Given that the unit of analysis being the county, the median income of the households within the county is commonly used as an estimate of the socioeconomic status for the population of the county. Since median income would vary by the socioeconomic status of the population of the county, it would allow us to compare the COVID incidence and mortality between “high income” counties versus “low income” counties. Together they answered our main study questions of “the impact of COVID-19 in the counties nationwide according to their socio-economic status and investigate whether the impact varies by counties of states with Medicaid expansion versus those without Medicaid expansion.”**

- “Did US have different Medicaid expansion for the high, medium and low wealth index?”

**R/ Excellent question. Medicaid provides a source of health insurance for low-income households and/or those with disability, among other factors. However, the income and qualification thresholds for enrollment into Medicaid differ by state, which depend on the state government’s policy. If a state has adopted the policy of Medicaid expansion, the requirements for Medicaid eligibility are less and at lower income thresholds, which makes it easier for people to enroll in health insurance versus states who did not adopt Medicaid expansion.**

- “How do you see the association between the income, Medicaid and COVID outcomes? If so let us take it as a sort of continuum of care, have you calculated the dropout rate per each level?”

**R/ Based on the above, it is possible that COVID incidence or mortality may disproportionately affect counties of lower socioeconomic status from states that did not adopt Medicaid expansion because less people in that state would have access to health insurance. Less access to health insurance can lead to worse COVID complications, such as death, because the access to and the continuum of, care, are impaired. Therefore, we first compared the COVID incidence and mortality amongst counties of different socioeconomic status (based on median household income) to study this association. Mortality rates were calculated for each quartile of the median household income. We then looked at the impact of Medicaid expansion on this association by comparing the COVID mortality amongst counties of different socioeconomic status within states that adopted Medicaid expansion and those that did not.**

- “Use scientific methods of writing as per the standard. There are inconsistencies. Language, tense, sentences, ideas, paragraphs and the general manuscript needs to be revised?”

**R/ We agree with the reviewer and have reviewed the manuscript to ensure the use of scientific methods of writing.**

- “Check the whole statistics again”

**R/ We appreciate your review of our work. We performed a thorough review of the statistics, we did not find any items in need of correction at this point.**

We appreciate and thank the reviewer for his/her comments, to which we provided a point-to-point answer in the reply to reviewer’s document, all of which, made our paper a much improved one.

Tsikata Apenyo & Wen-Chih Wu
