# Supplementary material for: Association between median household income, state Medicaid expansion status, and COVID-19 outcomes across US counties
Source: PLoS One. 2022 Aug 11;17(8):e0272497. doi: 10.1371/journal.pone.0272497 (PMC9371257; doi:10.1371/journal.pone.0272497)
Supplement: Attachment — Submitted filename: Response to reviewers-7-15-22.docx [file pone.0272497.s005.docx]

Author’s Reply to Reviewer’s Comments

We appreciate and thank the reviewers for their comments, to which we provided a point-to-point answer below:

Reviewer #3:

The revised manuscript is generally well-presented and presents an interesting topic. Many of my comments are minor. My biggest concern is that the authors proceeded with the outcome of crude mortality and crude incidence rate. I know that US counties are very diverse in terms of age distribution and as such, the age-adjusted mortality rates and age-adjusted incidence rates would have been the better metrics. Please provide enough justification for not age-adjusting rates. Having said that, I have seen plenty of studies examining the crude rate, primarily because of the lack of data. If that is the case, please provide this information in the limitation section.

**R/ We agree with the reviewer that age-adjusted mortality rates and age-adjusted incidence rates would have been the better metrics. However, the data available does not have age-adjusted rates for which we have added this as a limitation in the discussion section (pg. 13, second to last paragraph).** **We also stated that we adjusted for age, gender, race and comorbidities of the county population to minimize the residual confounding.**

Key findings /Questions: household income should be replaced with county-level median HH income.

**R/ “household income” replaced with “county-level median household income” in Key findings /Questions**

Abstract:

Objective: What you have as the objective in the abstract is not really an objective. That’s background information.

**R/ We replace the sentence with “To study the relationship between county-level COVID-19 outcomes (incidence and mortality) and county-level median household income and status of Medicaid expansion of US counties”**

Methods: Use the easy-to-understand word format for the date given the global readership of the journal. Avoid star; either use the proper multiplication sign or have it in word.

**R/ We changed the date format to “January-20th-2021 through December-6^th^-2021”. We deleted the star and changed it to “Medicaid-expansion-times-income-quartile interaction”**

Results: insert “rate” after COVID-19 incidence and COVID-19 mortality

**R/ “rate” was inserted after COVID-19 incidence and COVID-19 mortality through out to be read as “incidence-rate” and “mortality-rate”.**

Background: On your hypothesis, household income should be replaced with county-level median HH income.

**R/ We replaced “household income” with “county-level median household income” in the hypothesis**

Outcome: See my general comment on the top

**R/ “rate” was inserted after COVID-19 incidence and COVID-19 mortality through out to be read as “incidence rate” and “mortality rate” or “incidence and mortality rates”.**

Statistical Analysis:

For the sake of completeness, also mention that these income ranges are per annum.

“Using county population as the denominator in the model”: well there is no such thing as “denominator” in the model. Either you specify the rate (covid outcomes divided by denominator population) as the outcome, or most likely given the NB model, you specified the population as an offset. Make this clearer.

**R/ “Median household income” was changed to “County-level median household income per annum” under Main Exposure Variables. We also made the same change to “county-level median household income per annum” under the statistical analysis. We also changed the word “denominator” to “offset”.**

I see the value of having random intercepts for counties but not for the reason the authors suggest (i.e. to account for clustering, especially in conjunction with an unstructured covariance matrix). I would have used the robust standard errors clustered at the state level. But I am not holding this relatively small issue against the authors for their enormous effort.

**R/ We thank the reviewer for their understanding.**

“If the interaction proved to be significant”: note that you are not proving significance. I’d

rephrase this sentence.

**R/ the sentence was rephrased to “If the interaction was significant…”**

Table 1 title” Again, avoid giving an impression as if the income is household level. Even if the software gives you P=0, this is theoretically not possible. Write something like P<0.0001.

**R/ We have changed to “County-Level Median Household Income” for titles and sub-titles of the tables. We changed the P values=0 to P<0.0001.**

Thank you sincerely for the insightful comments which made our paper a much improved one.

Sincerely,

Tsikata Apenyo & Wen-Chih Wu
